# Machine Learning Model Analysis of Breeding Habitats for the Black-necked Crane in Central Asian Uplands under Anthropogenic Pressures

**DOI:** 10.1038/s41598-017-06167-2

**Published:** 2017-07-21

**Authors:** Xuesong Han, Yumin Guo, Chunrong Mi, Falk Huettmann, Lijia Wen

**Affiliations:** 1 College of Nature Conservation, Beijing Forestry University, P.O. Box 159, Beijing, 100083 China; 2 Institute of Geographic Sciences and Natural Resources Research, University of Chinese Academy of Science, Beijing, 100049 China; 3 EWHALE Lab-, Department of Biology and Wildlife, Institute of Arctic Biology, University of Alaska Fairbanks, 419 Irving I, P.O. Box 757000, AK, 99775 USA

## Abstract

The black-necked crane (*Grus nigricollis*) is the only alpine crane species and is endemic to the Tibetan Plateau. The breeding habitats of this species are poorly understood, which greatly hampers practical research and conservation work. Using machine learning methods and the best-available data from our 7,000-kilometer mega-transect survey and open access data, we built the first species distribution model (SDM) to analyze the black-necked crane’s breeding habitats. Our model showed that current conservation gaps account for 26.7% of its predicted breeding habitats. Specifically, the northern parts of the Hengduan Mountains and the southeastern Tibet Valley, the northern side of the middle Kunlun Mountains, parts of the Pamir Plateau, the northern Pakistan Highlands and the western Hindu Kush should be considered as its main potential breeding areas. Additionally, our model suggested that the crane prefers to breed in alpine meadows at an elevation over 2,800 m, a maximum temperature of the warmest month below 20.5 °C, and a temperature seasonality above 7,800 units. The identified conservation gaps and potential breeding areas can aid in clearly prioritizing future conservation and research, but more attention and study should be directed to the unassessed Western Development of China to secure this endangered crane lineage and other wildlife on the Tibetan Plateau.

## Introduction

**Figure 1:**
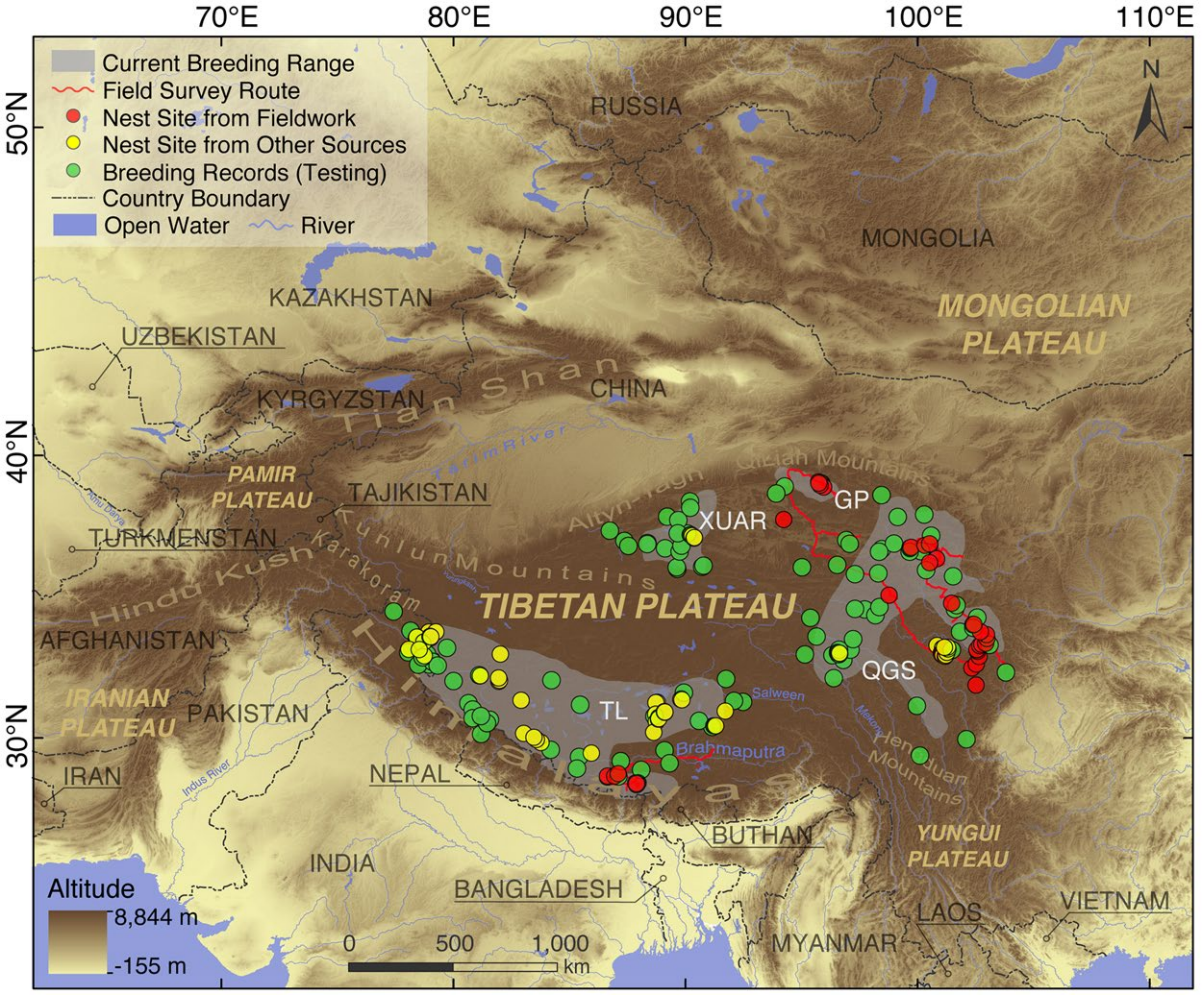
Study area, current breeding range, species distribution data, and field survey route. Presented on the digital elevation map (DEM) for displaying geographic conditions; the current breeding range was digitized by the author from Dorn Moore, International Crane Foundation (Li^9^). Detailed information for nest sites and breeding records is shown in Table 1. Field survey route (mega-transect) was recorded with a global positioning system (GPS) logger in real time. The map was made by the authors in ArcGIS 10.1 (ERSI co., USA) and processed in Adobe Illustrator CC (Adobe Systems Inc., USA).

The black-necked crane (*Grus nigricollis*; Taxonomic Serial Number 176187) is endemic to the wider Tibetan Plateau region in the Himalayas and is the only alpine crane species that breeds in the extensive landscape of high central Asia^1^. Owing to the plateau’s environmental inaccessibility to comprehensive and persistent field research, the black-necked crane remains the least studied crane species in the world^2^. At present, the International Union for Conservation of Nature (IUCN) classifies the black-necked crane as “vulnerable” because of its decreasing global population of 10,070–10,970 individuals^3^. However, the credibility of the references on which this assessment was based has been recently challenged by new continuous breeding records^4–7^. The black-necked crane shows the most restricted distribution in family Gruidae worldwide^8^, breeding exclusively in four separate areas: central and southwest Tibet and Ladakh (TL); parts of Qinghai, Gansu and Sichuan Provinces (QGS); northwest Gansu Province (GP); and the southeast corner of the Xinjiang Uygur Autonomous Region^9^ (XUAR; current breeding ranges, for distinction hereafter, CBRs; Fig. 1). However, the overall population count obtained from wintering grounds greatly outnumbers current estimates from these breeding grounds^10^. This discrepancy provides clear evidence that in the geographical continuum of the Tibetan Plateau, many surrounding alpine areas appear to be unstudied with regard to their existing crane population^11^.

This situation is especially important as human activities are increasingly affecting the Tibetan alpine wetlands overall, which provide indispensable habitats for the black-necked crane^8^. Perceived as the “Spiritual Bird” in Tibetan Buddhism, the crane has long lived under the conservation and even worship of local Tibetan nomads^10^ and has been labeled as “moderately tolerant” to human activities^2^. However, this judgment may be misleading and might lead to some degree of neglect in practical conservation schemes. Currently, as one of the least economically developed regions in China, the Tibetan Plateau holds a key position in the ongoing Western Development scheme of China and suffers greatly from aggravating environmental reform activities^12, 13^. Mineral exploitation and transportation, ongoing wetland degradation, the construction of infrastructure and changes in agricultural practices under the guidance of this policy are severely threatening the crane’s breeding habitats^3, 4, 9^, while many consequent threats to the black-necked crane are overlooked. Therefore, effective and powerful conservation measures are urgently needed to maintain unharmed habitat for the crane.

However, no monitoring, conservation or management activities can be effectively conducted without sufficient knowledge of the crane’s distribution^14^, especially the locations of its breeding habitats that directly contribute to the crane’s reproduction^10^. Owing to rugged environmental conditions and a low infrastructure density, traditional survey methods that rely on road networks or residential settlements cannot be easily applied for exploring the largely unpopulated areas in this multi-national alpine landscape. This limitation has become a serious bottleneck in the modern study and conservation of the black-necked crane. The deficiency of available information concerning the breeding distribution of this species is not an isolated case but, rather, represents a common situation for the avian fauna distributed and threatened in this area^15^. The black-necked crane is valued as an environmental indicator species and flagship species in Tibetan alpine wetland ecosystems^2, 16^. As such, we suggest that studying the crane’s missing breeding population and associated habitats can be used to infer suitable habitats for the majority of the water birds that breed in this extensive and understudied alpine landscape.

To this end, with the best-available species and environmental data compiled from our mega-transect survey and open access databases, we successfully built the first machine learning species distribution model (SDM) to predict the breeding distribution of the black-necked crane in multi-national central Asia uplands featuring 18 nations and an extensive alpine landscape. Based on the model predictions, we determined conservation gaps and potential areas for the black-necked crane’s breeding habitats and highlighted associated overlooked threats. Our aim is to present clear priorities for the next steps in the conservation and investigation of this globally threatened crane species, as well as other wildlife within the crane’s habitat.

## Methods

### Study area and field work

In view of the black-necked crane’s exclusive preference for alpine wetlands on the Tibetan Plateau^17^, we centered our research on the globally relevant alpine landscape in central Asia featuring 18 nations and five great plateaus. Within this geological continuum, the Tibetan, Pamir, Mongolian, Yungui and Iranian Plateaus are ecologically connected through many mountain ranges, including the Himalayas, the Hengduan Mountains, the Kunlun Mountains, the Karakoram, and the Hindu Kush. Consequently, considering the landscape continuity in this extensive region, we delimited the land within a bounding box of 20.24°N – 52.59°N, 62.09°E –111.79°E in ArcGIS 10.1 (ERSI co., USA) as our study area, comprising over 24,000,000 km^2^ (Fig. 1).

The black-necked crane is known to arrive at breeding areas in late March and is described to depart by late October^10^; accordingly, we conducted a 7,000-kilometer mega-transect road survey in the crane’s known breeding habitats in Qinghai, Gansu and Sichuan Provinces and the Tibet Autonomous Region, China (April – July, 2014; Fig. 1). During our fieldwork, alpine meadows along roads were searched within a 2-kilometer belt using 360-degree point scan methods (10-minute point count)^7, 18^. When nests were discovered, the coordinates of accessible nests were recorded with a global positioning system (GPS) logger; alternatively, inaccessible nests (e.g., isolated in a lake or swamp) were geo-referenced by locating their relative direction and distance to the observation spots (conducted with triangulation and cross location using Google Earth, https://earth.google.com/).

### Species distribution data and environmental variables

We obtained 183 black-necked crane nest coordinates from a combination of our own field work and from previous studies, covering all four of the CBRs (Table 1 and Fig. 1). Ambiguous records that did not provide breeding details could cause bias because they may represent occasional crane occurrences outside the breeding range, whereas our confirmed nest sites exclude such interference (floaters). SDMs must be assessed through empirical distribution data of species^14, 19^; therefore, we referenced 102 additional breeding records from previous research as testing points for model validation (Fig. 1 and Table 1).

**Table 1:** Detailed Distribution Data and Sources.

Location	Count	Time	Source	Usage
Yanchiwan (GP)	22	2014	Fieldwork^27^	Training
Eastern Qinghai (QGS)	22	2014	Fieldwork^27^	Training
Zoige (QGS)	14	2014	Fieldwork^27^	Training
Pumqu Basin (TL)	17	2014	Fieldwork^7, 27^	Training
Nyanpo Yuzee (QGS)	14	2012	Tashi Sangpo, unpublished data	Training
Ladakh (TL)	15	1983–2012	Published Research^52^	Training
Longbao (QGS)	29	2012	Published Research^53^	Training
Altyn-Tagh (XUAR)	13	2012	Published Research^12^	Training
Ngari (TL)	6	2014	Wang & Li, unpublished data	Training
Central Tibet* (TL)	31	1992	Published Research^54^	Training
Overall Breeding Range	82	1978–2001	Published Research^2^	Testing
Altun Reserve (XUAR)	18	2012	Published Research^55^	Testing
Mapangcuo (QGS)	1	2012	Published Research^5^	Testing
Haizishan (QGS)	1	2013	Published Research^6^	Testing

Nests shown in maps were referenced in ArcGIS 10.1 (ERSI co., USA) to obtain a decimal longitude and latitude with five decimals (using the Georeferencing tool in ArcGIS). In the first column, “TL” represents central and southwestern Tibet and Ladakh; “QGS” represents part of Qinghai, Gansu and Sichuan Provinces; “GP” represents northwestern Gansu Province; and “XUAR” represents the southeast corner of Xinjiang Uygur Autonomous Region. *Nest locations presented were only accurate to the geographic minute in the literature (two sites thus overlapped).

Twenty-nine environmental variables (predictors) were downloaded from public open access databases (Supplementary Table S1) and were then used for model construction in Salford Predictive Modeler v7.0 (SPM; http://www.salford-systems.com/; Salford Systems Ltd., USA; a time-limited free version of SPM is provided online for trial). Specifically, our predictor set included six geological variables (altitude, slope, aspect, distance to lakes, distance to rivers and distance to coast), three anthropogenic variables (distance to roads, distance to railroads and distance to settlements), 19 bioclimatic variables (WorldClim, http://www.worldclim.org/; derived from climate data for the period 1960–1990)^20^, and land cover class (Supplementary Tables S1 and S2).

### Species distribution model (SDM) development

Our workflow for Random Forest GIS model predictions conceptually followed the research of Kandel *et al*. (2015) in the Hindu-Kush Himalaya Region^21^. Using the Geospatial Modeling Environment (GME; http://www.spatialecology.com/gme/), we selected 18,300 pseudo-absence points by random sampling across the study area at a ratio of 1/100 (presence/absence) to provide representative landscape samples across the vast Himalaya region^14^. To maintain our model’s applicability to practical investigation and conservation actions^22^ and considering our predictor layers’ resolution (300 m – 1,000 m; Supplementary Table S1), in this study, we set the resolution to 1,000 m. Using the WGS 1984 Mercator projection, we generated a total of 24,718,388 background lattices (a 1,000 m-spaced point grid data set) in the study area. We then extracted the habitat information from 29 environmental layers for all of the background lattice, presence, pseudo-absence and testing points.

As an ensemble classification and regression tree algorithm, Random Forest is considered among the group of leading data-mining machine learning methods for its high accuracy in ecological predictions^23^. Further, it features great tolerance to noise^24^, a strong immunity to overfitting^24^ and high efficiency in processing a large number of predictors and their interactions^14, 25^. Owing to “recursive partitioning” and bagging, SPM algorithm optimization can properly handle interactions, stopping rules, weighting and complexities in predictor combinations^24–27^. Two modifications were applied in the model settings: we used balanced class weights, a powerful and sophisticated weighting function in SPM, to defend against inequivalent prevalence (183 presence versus 18,300 absence points)^27^; we set the number of trees to 1,000 to find the best possible model^27, 28^. Other settings in SPM were left as default.

As a next step, in SPM, we used the built model (grove file) to predict (“score”) the crane’s occurrence possibility in each lattice and then mapped out the resulting spatially referenced dataset in ArcGIS using inverse distance weighted (IDW) methods for visualization^27, 28^. Eventually, we obtained a continuous interpolated prediction map in which each lattice was assigned with the relative index of occurrence (RIO) of the breeding black-necked crane (ranging from 0 for predicted absence lattices to 1 for predicted presence lattices). We then generated variable importance rankings and associated response curves to reveal the correlations between species occurrence and environmental variables (see Supplementary Table S1 and Supplementary Fig. S2). This was done by building a tree-based model (TreeNet) in SPM to mimic a Random Forest run^21^ (as suggested by Salford System, Ltd.). For the following case studies, we further created a binary presence/absence prediction map with a threshold at 0.58, which was calculated from the threshold table according to the sensitivity-specificity sum maximization approach (the threshold table was generated in SPM; see Supplementary Table S3 for justification)^29^.

### Model assessment and validation

In this study, we applied three criteria to assess our model’s performance on the training data. First, we used the two prevalence-independent indices: the receiver operating characteristic curve (ROC curve, expressed as the area under the curve, AUC) to test the model’s discrimination ability^30^, and the true skill statistic (TSS) as a supplementary measure for the model’s presence/absence prediction^31^. The AUC value was obtained by using the out-of-bag (OOB) predicted occurrences for each record, and the TSS value was calculated from the threshold table generated by Random Forest in SPM (Supplementary Table S3)^31^. As a next step, to confront the model predictions with real species distribution data from an ecological perspective^19^, we extracted RIOs from the continuous prediction map for 102 testing points and then analyzed the model’s fit using boxplots with 95% confidence intervals. These testing points represent real breeding distribution sites of the black-necked crane and were not used to train the model; therefore, the comparison between their predicted RIOs and value “1” (real presence) can be used to indicate the model’s performance in undersampled areas^27^.

### Conservation gap analysis

To provide an ecologically meaningful analysis of the conservation gaps of the black-necked crane’s breeding habitats, we used (i) the World Database on Protected Areas (http://www.protectedplanet.net/) to infer an area’s conservation status^32^ and (ii) the Human Influence Index (HII; http://sedac.ciesin.columbia.edu/) to infer an area’s human population status^33^. We used HII because extensive unpopulated areas located in this region remain ecologically primitive and therefore should be excluded from conservation gaps, which are generated for revealing the priorities for future conservation efforts^32^. In the HII, human influence ranges from 0 (no human influence) to 64 (severe human influence)^33^. To infer whether the black-necked crane’s breeding habitats were threatened by human disturbances, we reclassified HII with a five-unit interval and then selected a conservative threshold (“safety cut-off”) of HII = 10 based on expert opinions, where the HII map was most consistent with the actual vulnerable areas indicated in previous studies^1, 8, 9^. This threshold was additionally tested according to our knowledge of regional human influences from our empirical fieldwork. By overlaying the binary predictions with existing protected areas and the reclassified HII map, we eventually selected predicted breeding habitats (RIO ≥ 0.58) that were threatened by human disturbances (HII ≥ 10) and that were located outside of protected areas as the conservation gaps for the crane’s breeding habitats.

### Potential breeding area determination

**Figure 2:**
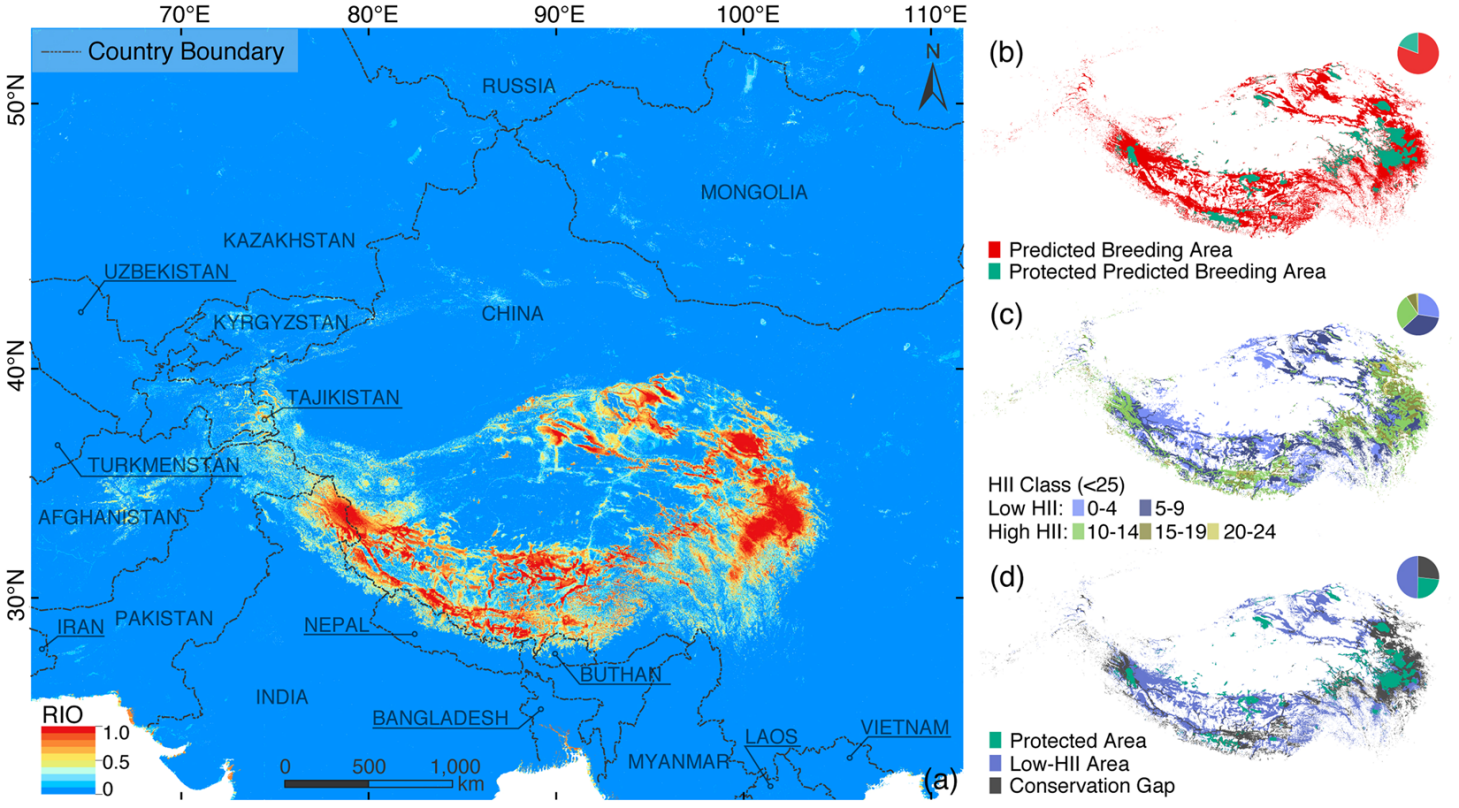
Prediction map, conservation status, anthropogenic pressures and conservation gaps. This map shows the black-necked crane’s (**a**) continuous prediction map in the study area, (**b**) conservation status analysis in predicted presence areas (RIO ≥ 0.58; binary prediction map overlaid with protected areas), (**c**) anthropogenic pressure analysis in predicted presence areas (RIO ≥ 0.58; map of Human Influence Index, HII) and (**d**) conservation gaps in its predicted presence areas (RIO ≥ 0.58). The maps were made by the authors in ArcGIS 10.1 (ERSI co., USA) and processed in Adobe Illustrator CC (Adobe Systems Inc., USA).

We determined the potential breeding habitats for the black-necked crane based on (i) our model’s binary prediction map, (ii) CBRs and (iii) collected breeding records (183 presence points and 102 testing points). As a first step, we maintained areas with RIOs over 0.58 in the study area as the base layer for potential breeding habitat delineation (Fig. 2b). From these pixels, we then excluded places that contained a reported presence of breeding cranes. Specifically, to leave a reasonable space for the detected breeding crane’s activity, and to provide a relatively conservative picture of the potential areas, we generated 67-kilometer buffer zones for CBRs and 285 overall breeding records (Supplementary Table 1; buffer distance was determined according to the crane’s dispersal capacity during the breeding season^10^). All of the predicted presence pixels (RIO ≥ 0.58) within this range were removed from the base layer. To transform the remaining spatially discrete pixels into a conservation- and management-operable map, we selected and aggregated those clustered pixels as major potential breeding areas for the black-necked crane (using the “aggregate points” tool in ArcGIS). Furthermore, to help locate the focal regions in the crane’s potential breeding areas and to then prioritize practical field surveys and relevant conservation design, we additionally calculated the densities of these predicted presence pixels located in the determined major potential areas (realized by transforming the pixels into points and then by using the “point density” tool in ArcGIS).

## Results

### Prediction map and assessment

The continuous and binary (RIO ≥ 0.58) prediction maps are respectively shown in Fig. 2a and b. From the ROC curve, our Random Forest model showed an AUC value of 0.995, indicating that it accurately captures the correlation between the black-necked crane and its breeding habitats (Supplementary Fig. S1), as values over 0.9 indicate excellent model discrimination^30^. Accordingly, in the presence/absence predictions, the TSS value was calculated to be 0.947, additionally supporting the Random Forest model’s performance on the training dataset (Supplementary Table S3). The boxplot for RIOs extracted from 102 testing points showed a median of 0.802 (showing 95% confidence intervals), suggesting that our model could accurately predict the crane’s empirical breeding habitats (Supplementary Fig. S2)^27^.

### Variable importance rankings and response curves

Obtaining good predictions is the prime goal in machine learning^24^, and mining the dominant predictors and understanding their influencing mechanisms are also important steps in applying efficient conservation schemes^14^. Variable importance rankings indicated that elevation (Altitude, score: 100.0), maximum temperature of the warmest month (Bio_5, score: 99.3), temperature seasonality (Bio_4, score: 50.0) and land cover class (Landcv, score: 45.1) were the four most important environmental variables in the black-necked crane’s breeding habitat preference. Correspondingly, their response curves of these variables suggested that, within the study area, the crane tends to breed in herbaceous plant-covered sites at an elevation above 2,800 meters, a warmest monthly maximum temperature below 20.5 °C and a temperature seasonality greater than 7,800 units (Supplementary Fig. S3). The complete variable importance rankings and their associated response curves are shown in Supplementary Table S1 and Supplementary Fig. S3.

### Conservation gap analysis

The overlay analysis for conservation status showed that, within the study area, only 23.6% of the predicted breeding grounds were located in protected areas (Fig. 2b). Alternatively, the reclassified HII map indicated that, in general, the overall anthropogenic pressures on the predicted breeding area of the black-necked crane were rather low: 98.7% of the area showed an HII under 20, and 63.0% of the area was mostly free from human disturbances (HII < 10; Fig. 2c). Among the sites under relatively strong anthropogenic pressures (the remaining 37.0% of the area with HII ≥ 10), only 30.7% were protected areas (Supplementary Table S4). The overall distribution of the unprotected breeding habitats and high HII areas showed a spatial consistency on the eastern Tibetan Plateau, the northern Hengduan Mountains, the middle and lower reaches of the Brahmaputra River, and western Karakoram (see Fig. 1 for geography). Consequently, we designated these places as the conservation gaps for the breeding black-necked crane, accounting for 26.7% of its overall predicted breeding areas (Fig. 2d).

### Current breeding ranges (CBRs) and potential breeding area

**Figure 3:**
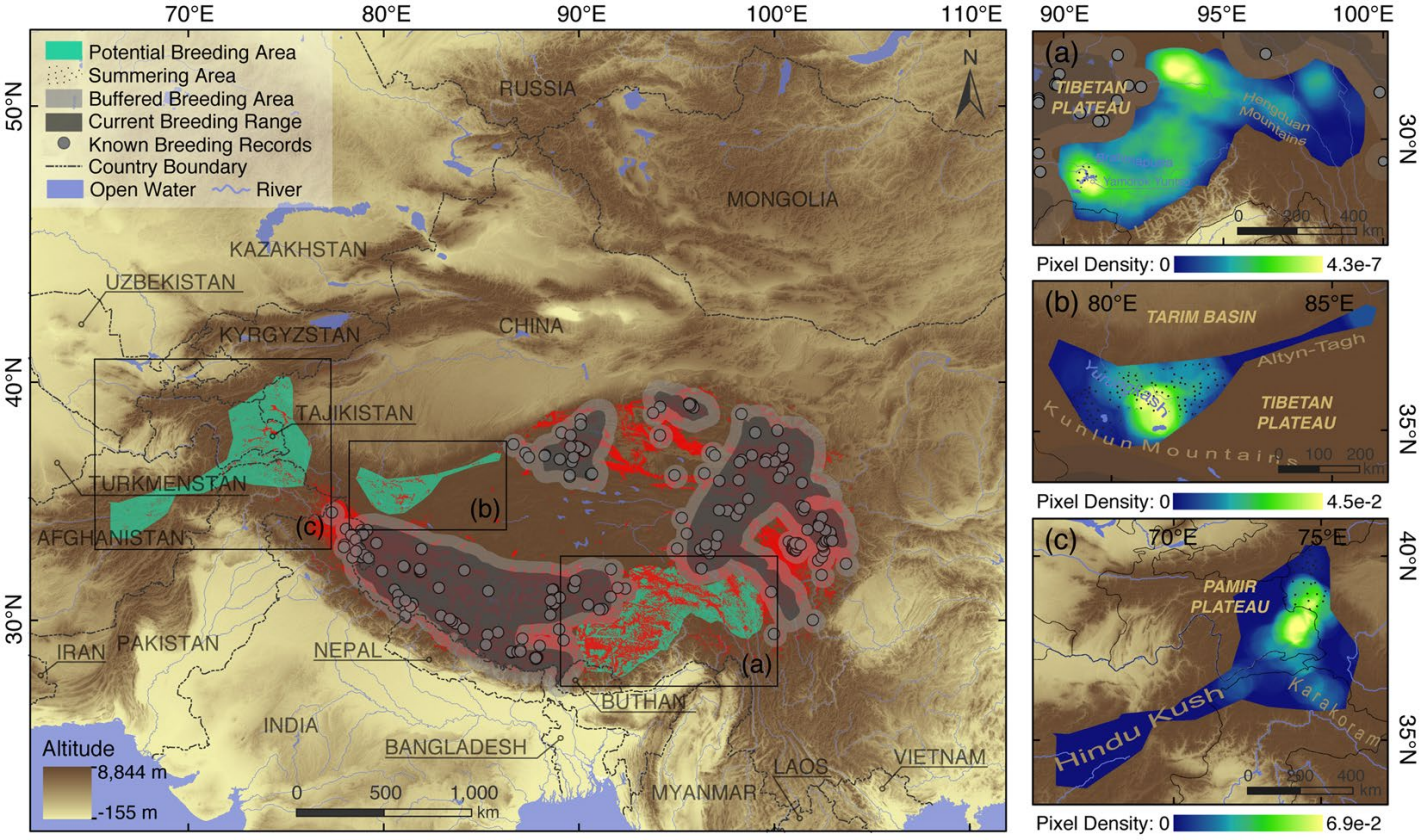
Map of potential breeding area. The map shows the potential breeding habitats of the black-necked crane, including (**a**) the northern parts of the Hengduan Mountains and the southeastern Tibet Valley; (**b**) the northern side of the middle Kunlun Mountains; and (**c**) parts of the Pamir Plateau, the northern Pakistan Highlands and the western Hindu Kush. The maps were made by the authors in ArcGIS 10.1 (ERSI co., USA) and processed in Adobe Illustrator CC (Adobe Systems Inc., USA).

The binary prediction map showed that the breeding habitats of the black-necked crane are mostly distributed along the edge of the Tibetan Plateau, and four CBRs are not isolated but connected by continuous suitable breeding habitats (Fig. 3). Specifically, CBRs covered 52.5% of the crane’s predicted breeding habitats (RIO ≥ 0.58), whereas approximately 60.3% of the area of CBRs was predicted to be unsuitable for the black-necked crane to breed (Fig. 1). Out of the buffered CBRs and breeding records, a total of 158,138 pixels were predicted to show high suitability for the cranes to breed (RIO ≥ 0.58), and these occurred precisely where new breeding records were recently documented^4–6^. Among them, on the southeastern, northwestern and western fringes of the Tibetan Plateau, three geographic zones showed clustered high-RIO pixels, and we therefore delineated (a) the northern parts of the Hengduan Mountains and the southeastern Tibet Valley (Fig. 3a,b) the northern side of the middle Kunlun Mountains (Fig. 3b and c) parts of the Pamir Plateau, the northern Pakistan Highlands and the western Hindu Kush (Fig. 3c) as three major potential breeding areas for the black-necked crane, respectively accounting for 46.5%, 1.4% and 3.2% of the overall uncovered predicted presence pixels. Moreover, pixel density further indicated that the Yamdrok Yuntso and the northern Hengduan valleys, the Yurangkash Basin, and the southeastern Pamir Plateau should be respectively considered as priorities for field investigations in the three potential areas identified above.

## Discussion

### Identified conservation gaps and limitations

In this study, anthropogenic pressures were represented by HII, and these were then used as an alternative criterion to identify conservation gaps for the black-necked crane’s breeding habitats. In contrast to common practices that simply spatially overlay biodiversity hotpots or focal species distributions with existing reserve networks and other land management categories (see Jennings, 2000 for a review)^32^, we excluded those conservation-lacking but unaltered habitats from our resulting gaps because in these areas, industrialization and globalization are still absent and the balanced relationships between humans and nature are maintained as in past millennia^11^. We therefore propose that this approach helps to identify for the public those conservation gaps that are more pertinent to the original intention of the conception of conservation gaps, “a way to set priorities for the next steps of conservation actions”^34^. Otherwise, a 76.4% conservation gap in the crane’s breeding habitats, which are located in extensive central Asia uplands, could hardly provide any valuable information and would rather serve as an exaggerated index and a broad map lacking focus and priority.

Consequently, it is clear from our predictions that most identified conservation gaps are located in the regions surrounding existing protected areas (Fig. 2d). Such a spatial concentration allows us to cover these conservation-gap areas more effectively by expanding existing protected areas. However, the result of “26.7% conservation gaps” may be referred to as a conservative estimate because of the incompleteness of the HII. Despite the best available data for measuring anthropogenic pressures in the study area, the intrinsic time scale of HII (1994–2004) has probably been outdated for over one decade^33^. During this period, the crane’s habitats have witnessed rapid development – the overall gross domestic product (GDP) of industry across the five administration regions increased by 356.7%^35^. Such economic growth can only be established on aggravating environmental reforms, and these have not been captured by the HII. Under the implementation of China’s Western Development scheme, many critical but unassessed human activities are pervasive in the breeding habitats of the black-necked crane. However, deficient knowledge regarding these threats and their links to this national policy are still widely overlooked, which greatly constrains current research and regional conservation activities.

For example, in western China, the breeding habitats of the black-necked crane are commonly fragmentized by fences^1^, which prevent chicks from escaping from predation by Tibetan foxes (*Vulpes ferrilata*) or widespread shepherd/feral dogs (observed during our fieldwork in Zhaguo, Tibet). In the middle and lower reaches of the Brahmaputra River (TL), highland barley was traditionally planted continuously as a method of protecting against famine and as a symbol of wealth^10^. However, the improvement of the governmental welfare system changed the land from crane-edible barley plantation to crane-unbeneficial but highly profitable cash crops, such as rapeseed (the sown area increased by 186.9% in 2005 compared with that in 1987)^36, 37^. The direct influence of booming tourism on the breeding grounds of northern and southern QGS has already been comprehensively studied by previous research^9^, yet its subsequent chain reactions and synergies remain overlooked, among which the massive Han Chinese migration influx for tourism and affiliated infrastructure construction is especially serious^35, 38^. We propose that great attention should be paid to the imperceptible but virtually fatal impact of their secular lifestyle on the ecological recognition and conception of Tibetan Buddhism, which has sheltered local wildlife and associated habitats over the past millennia^39^.

### Major potential breeding areas and conservation status

Based on the model’s binary prediction and collected breeding information, we identified potential breeding habitats for the black-necked crane, and we selected the following three regions as its major potential breeding areas for special consideration.

The northern parts of the Hengduan Mountains and the southeastern Tibet Valley were predicted to possess concentrated high-RIO pixels, accounting for 46.5% of all the uncovered predicted presence pixels (with RIOs over 0.58 and located out of buffered breeding ranges; Fig. 3a). The Hengduan Mountains are located on the eastern fringe of the Tibetan Plateau, consisting of a series of longitudinally arranged mountains and rivers (see Fig. 1 for geography). Liu *et al*. (2012) suggested that wetlands of this region are widely used as stopover sites by migrating black-necked cranes^5^ and that they also possess similar environmental characteristics to the cranes’ breeding habitats (such as elevation, habitat and vegetation types)^40^. Such similarity in environmental features may provide fundamental conditions for the crane to breed here. The southern Tibet valley is situated in the middle and lower reaches of the Brahmaputra River, and traditionally, this region is known to be among the crane’s most important wintering grounds^9, 37^. However, in late July 2014, we surveyed this region and recorded four adult black-necked cranes in the Yamdrok Yuntso and 111 adults (including 17 breeding pairs accompanied by chicks) in the Pumqu Basin^7^ (Fig. 3a). Considering the current tendency that Tibetan water birds appear to breed in their wintering grounds^41^, these records suggested that the black-necked crane may also show a similar breeding inclination in its winter grounds in this region. This region is the most promising potential breeding area for the black-necked crane, but currently, it is also the most threatened one. In the latest version of Western Development’s “12th Five Year Plan,” the Tibetan Autonomous Region is listed as the only exception in the directive for reducing energy consumption^13^. Furthermore, this region is assigned as the core region for the next stage of the large-scale construction of water conservancy projects – nine large water power basements and plants are scheduled or already constructed in the Brahmaputra and Mekong Rivers, which are located precisely in the center of the potential breeding area we delineated^42^.

The northern side of the middle Kunlun Mountains is located in the southern fringe of the Tarim Basin, where rivers such as the Yurangkash flow (Fig. 3b; see Fig. 1 for geography). Except for a compact cluster of high-RIO pixels (2.8%), a large-scale summering population of the black-necked crane (non-breeding population during the breeding season)^4^ also shows that this region is likely to constitute breeding habitat for the black-necked crane. Ma *et al*. (2011) recorded 60–90 individuals summering in the Yurangkash and neighboring river basins^4^ (Fig. 3b), and it is reported that the summering black-necked crane usually wanders within the breeding habitats or surrounding areas with similar environmental characteristics^17^, suggesting that this site possesses the fundamental conditions for the crane’s breeding. However, because of the generally arid climate, local settlements are primarily concentrated in oases along rivers, seriously conflicting with the black-necked crane’s potential breeding habitats. Moreover, in contrast to the Tibetan nomads, the hunting traditions that prevail among the local Uygur people also severely threaten the survival of cranes and other wildlife^43^.

Parts of the Pamir Plateau, the northern Pakistan Highlands and the western Hindu Kush make up a complex multi-national alpine landscape on the boundaries of China, Tajikistan, Afghanistan and Pakistan (Fig. 3c). In this region, the Northern Pakistan Highlands are located at the intersection of the Karakoram, the Hindu-Kush and the Himalayas. Among these features, the Hindu-Kush spreads westwards into Afghanistan and then extends into an extensive alpine region (the Iranian Plateau). Ecologically, this geological region is connected with the crane’s breeding grounds in Ladakh through the Himalaya and the Hindu-Kush, and it was predicted to show a concentrated assemblage of high-RIO pixels (3.2%). The black-necked crane has been recorded to summer in the eastern Pamir^44^. However, because of the rugged environmental conditions and complicated regional political conflicts, very limited further field investigations have been performed, and avian distribution information and data for this area are still widely lacking^4, 15^. In recent decades, this area has also been deeply affected by human activities: frequent military activities in Afghanistan, rampant terrorism, and an ongoing and massive Afghan refugee flow into northern Pakistan. Moreover, traditional hunting is still pursued by the people in the region, leading to a prevailing poaching pressure on the birds. Large migrating birds, such as cranes, easily become the dominant victims of this custom^45–48^. It is clear from our work that more attention should be paid to this region, and international efforts are desperately needed to investigate and improve conditions for birds there overall.

Therefore, for future on-the-ground investigations planned in the above major potential breeding areas, we propose to use our pixel density result as a primary guide for locating focal and prior regions and then to identify the crane’s fine-scale breeding distributions from our model predictions (1,000 m)^22^. Moreover, out of the three above identified areas, another 77,319 pixels were also predicted with RIOs over 0.58 and were not covered by buffered CBRs and breeding records (49.8%). Owing to their relatively dispersed distribution or proximity to known breeding grounds, we did not select them as major potential breeding areas that were prepared for special field actions. However, because of their locations at the margins of currently known breeding grounds, we propose that present research and conservation efforts could be strengthened and spatially expanded to cover these regions and eventually to determine the crane’s true breeding status.

### Breeding habitat preference

Among the 29 environmental variables tested here, elevation (Altitude) showed the greatest importance in the breeding habitat selection of the black-necked crane (Supplementary Table S1). Compared to subsequent predictors, its prominence likely results from the crane’s unique evolutionary history: the speciation of the black-necked crane occurred along with the uplift of the Tibetan Plateau and glaciation events, ultimately forming its current plateau-restricted distribution^49, 50^. This may also help us to interpret the crane’s exclusive preference for herbaceous plant-covered sites (Landcv), i.e., alpine meadows located in the study area. Bioclimatic predictor performance showed that temperature contributes more than precipitation: seven bioclimatic predictors of the top ten were temperature correlated, especially the maximum temperature of the warmest month (Bio_5) and temperature seasonality (Bio_4). The warmest month in the Tibetan Plateau occurs during the most critical period of the crane’s breeding season^10^, and temperature seasonality regulates most of the key ecological factors such as summer soil water content, moisture biological availability, and plant growing season duration and distribution^51^, all of which influence environmental suitability (especially food availability) for breeding cranes. Anthropogenic predictors (Distance to road, Distance to railroad and Distance to settlement) showed a limited importance in the crane’s breeding habitat selection (Supplementary Fig. S2). We think that this may be because (1) the black-necked crane shows a co-evolutionary “moderate tolerance” to local nomads and their traditional activities^2^, while (2) some more correlative modern anthropogenic pressures are not fully assessed. Again, we propose that this topic should receive more study and data collection should be undertaken for adequate wildlife research and management in this region.

In summary, for the first time, we used the best-available distribution data and leading machine learning methods to provide an accurate prediction of black-necked crane breeding habitats in the extensive and complex uplands of central Asia. The recovered prediction-based conservation gaps and potential breeding areas provide the global community with clear priorities for next-step conservation and investigation actions for this species. Above all, we propose that unassessed threats and their links to the Western Development of China and globalization must be specified and assessed for the long-term maintenance of this endangered crane species as well as for other wildlife and unique habitats on the fragile Tibetan Plateau.

## Electronic supplementary material

Supplementary Information
