# Supplementary material for: Machine Learning Model Analysis of Breeding Habitats for the Black-necked Crane in Central Asian Uplands under Anthropogenic Pressures
Source: Sci Rep. 2017 Jul 21;7:6114. doi: 10.1038/s41598-017-06167-2 (PMC5522491; doi:10.1038/s41598-017-06167-2)
Supplement: Supplementary file 1 — Supplementary Information [file 41598_2017_6167_MOESM1_ESM.pdf]

SUPPLEMENTARY INFORMATION:

Machine Learning Model Analysis of Breeding Habitats for the Black-necked Crane in Central Asian Uplands under Anthropogenic Pressures

Xuesong Han[a], Yumin Guo[a]*, Chunrong Mi[a,b], Falk Huettmann[c], Lijia Wen[a]

[a] College of Nature Conservation, Beijing Forestry University, P.O. Box 159, Beijing 100083, China;

[b] Institute of Geographic Sciences and Natural Resources Research, University of Chinese Academy of Science, Beijing 100049, China;

[c] -EWHALE Lab-, Department of Biology and Wildlife, Institute of Arctic Biology, University of Alaska Fairbanks, 419 Irving I, P.O. Box 757000, AK 99775, USA

Contact Information:

Xuesong Han: xuesonghan@bjfu.edu.cn

Yumin Guo*: guoyumin@bjfu.edu.cn (Corresponding Author)

Chunrong Mi: michunrong123@qq.com

Falk Huettmann: fhuettmann@alaska.edu

Lijia Wen: jiagefaner@126.com

Supplementary Information

Supplementary Figures S1-S3

Supplementary Tables S1-S4

No additional reference used.

Supplementary Figure S1. Boxplot for 102 testing points.

The plot was generated in R (http://www.r-project.org/), it showed 95% confidence intervals.

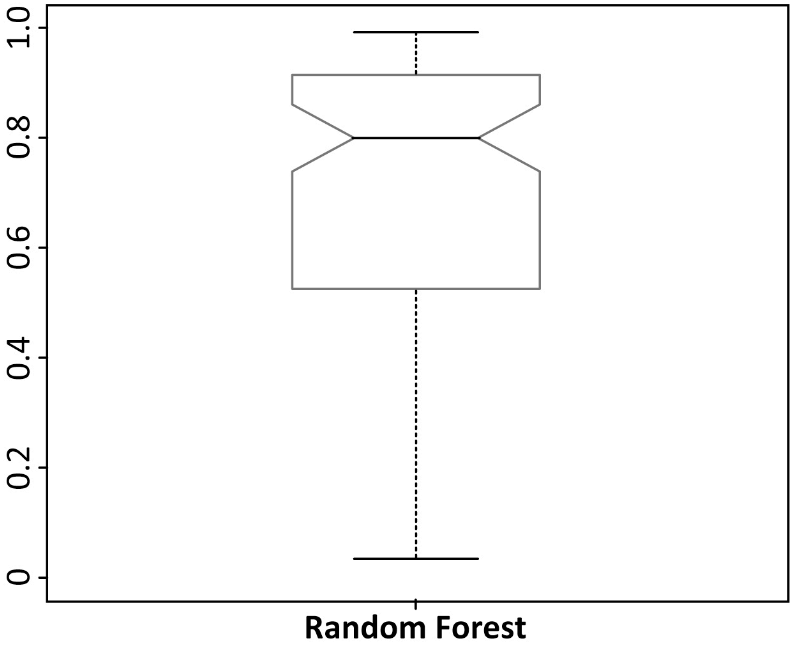

Supplementary Figure S2. Receiver operating characteristic (ROC) curve.

The area under the ROC curve (AUC) expressed as a proportion of the total area of the unit square defined by the false positive and true positive axes. AUC ranges from 0.5 (no discrimination ability) to 1 (perfect discrimination ability).

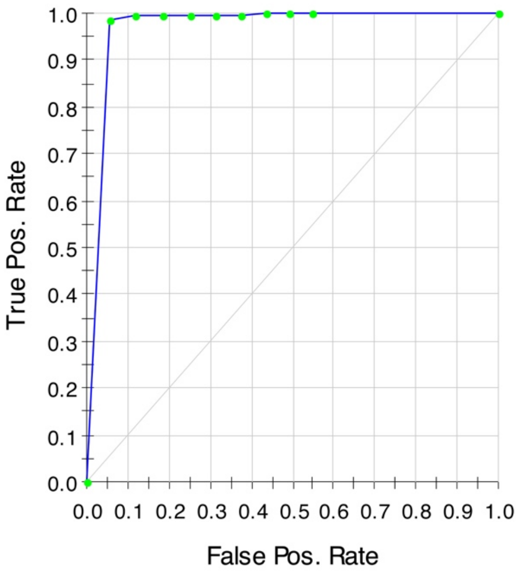

Supplementary Figure S3. Response curves for predictors.

The plots were generated in SPM v7.0 by TreeNet, it explained the correlation between the Black-necked Crane's occurrence and the environmental variables; three environmental variables (Bio_10, Bio_14, Slope) were ran as predictors of no importance (score: 0.000) in TreeNet, thus no response curve provided.

1. Altitude

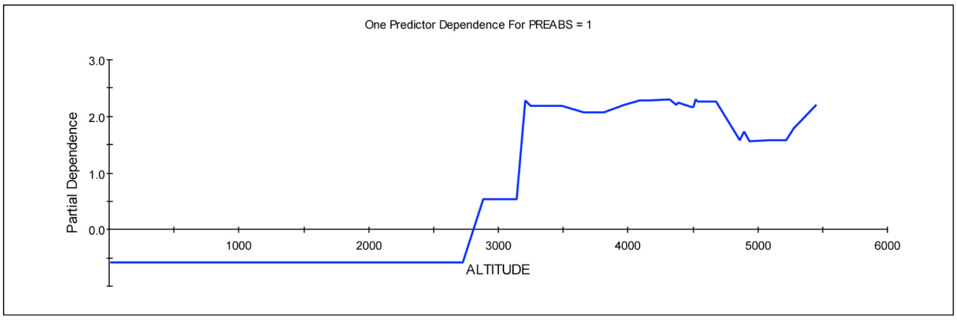

2. Aspect

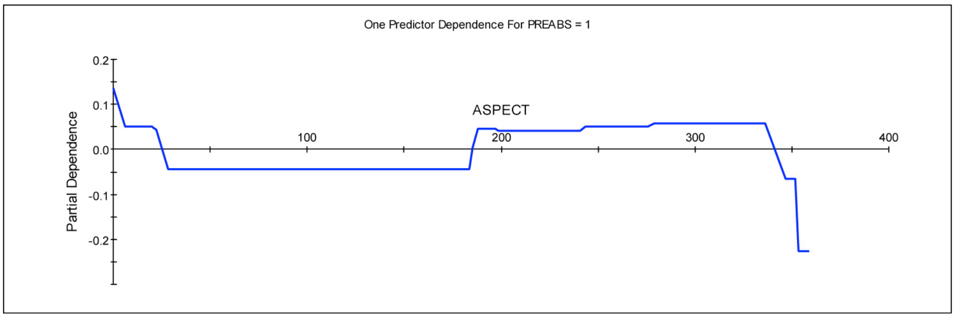

3. Bio_1

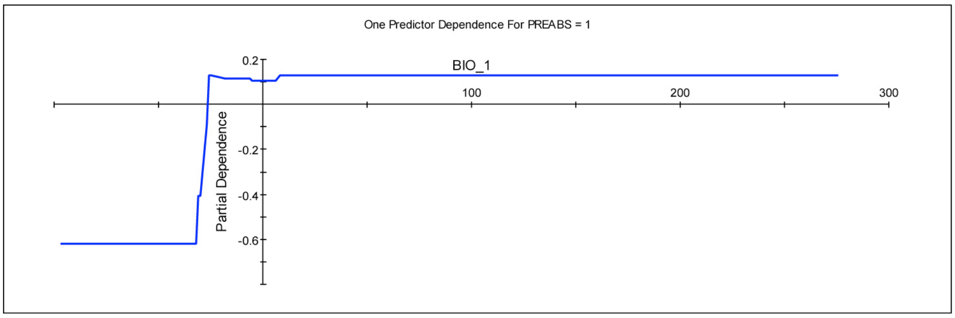

4. Bio_2

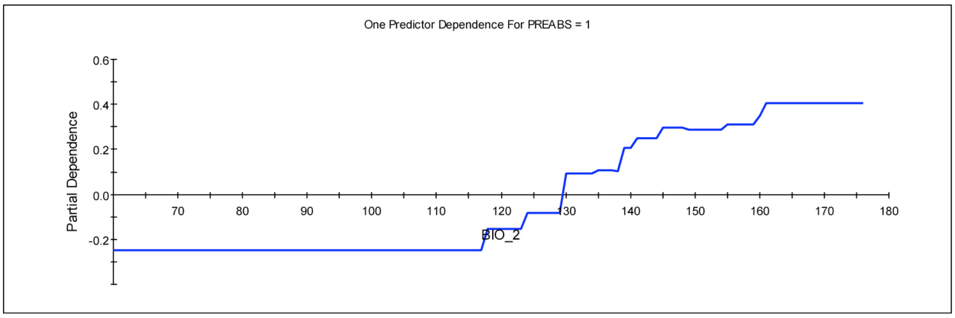

5. Bio_3

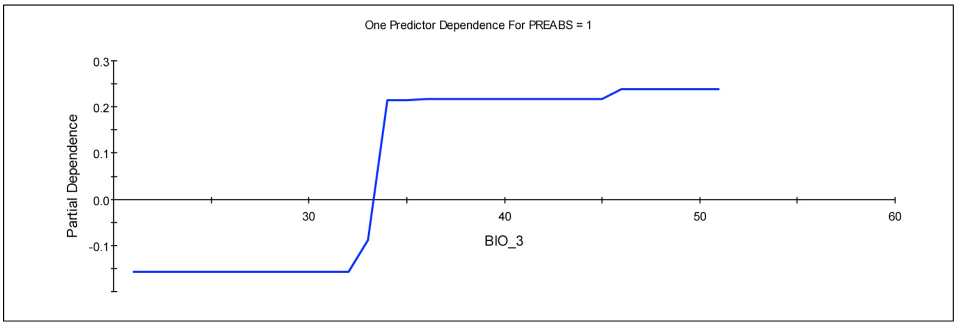

6. Bio_4

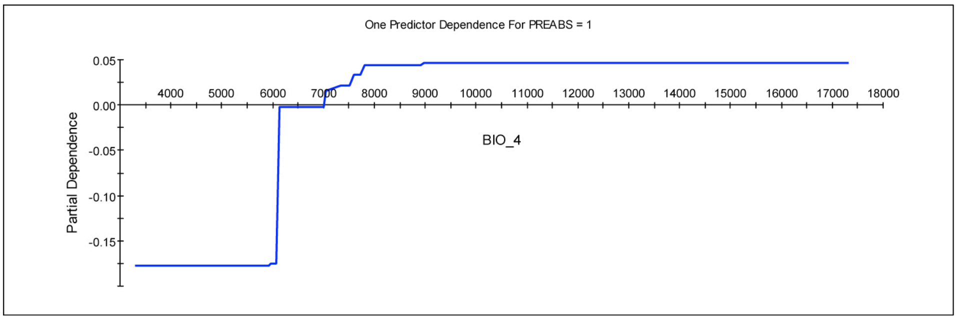

7. Bio_5

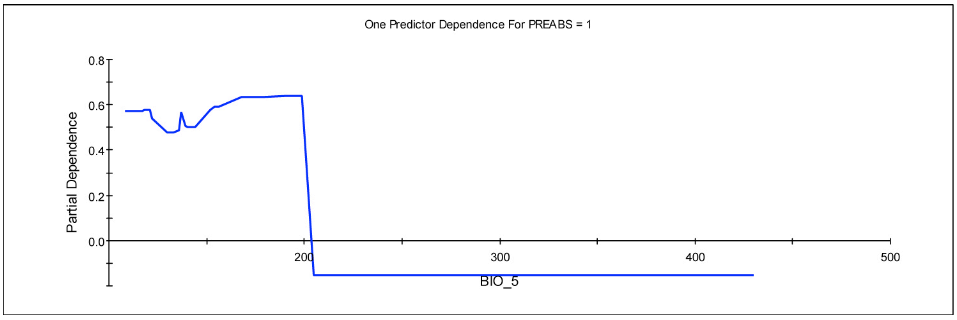

8. Bio_6

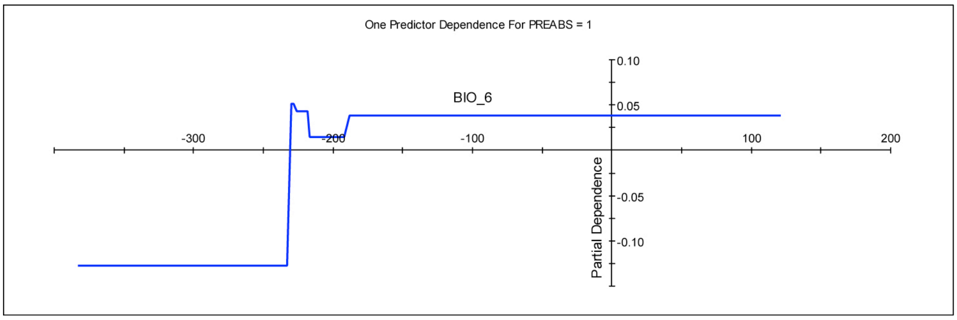

9. Bio_7

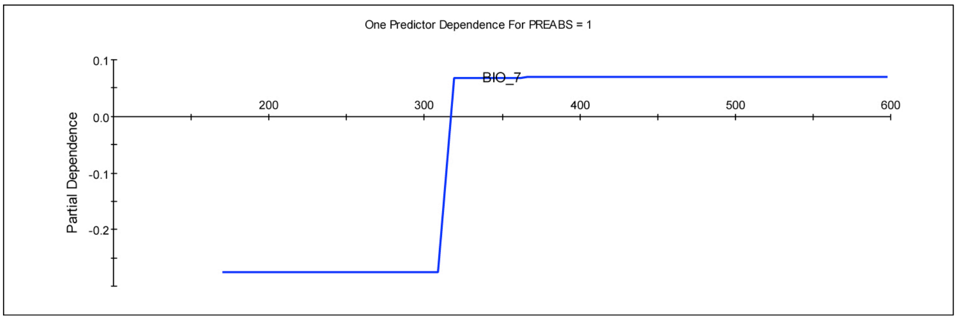

10. Bio_8

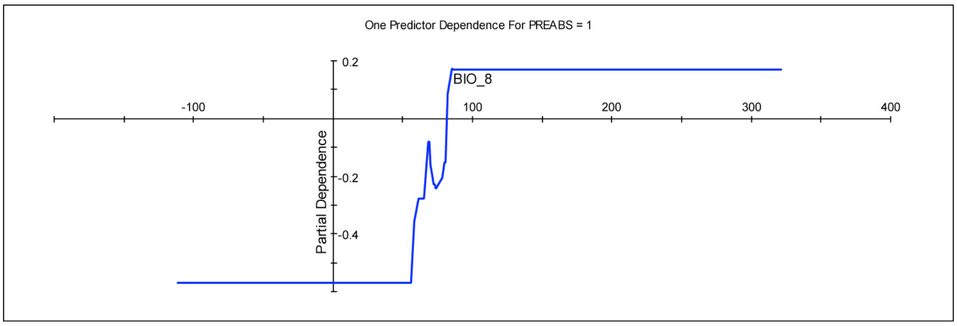

11. Bio_9

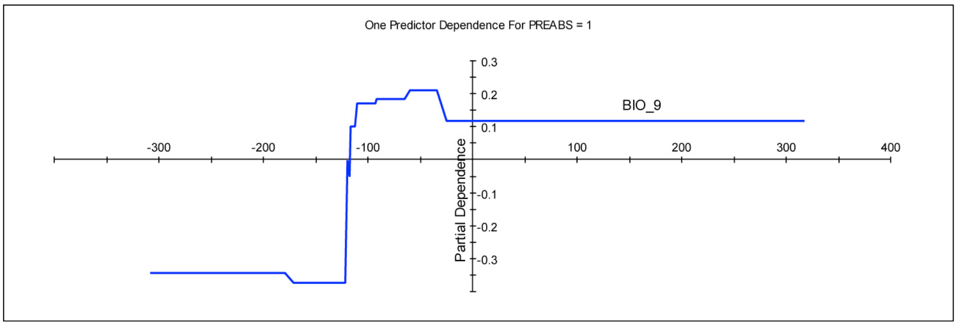

12. Bio_11

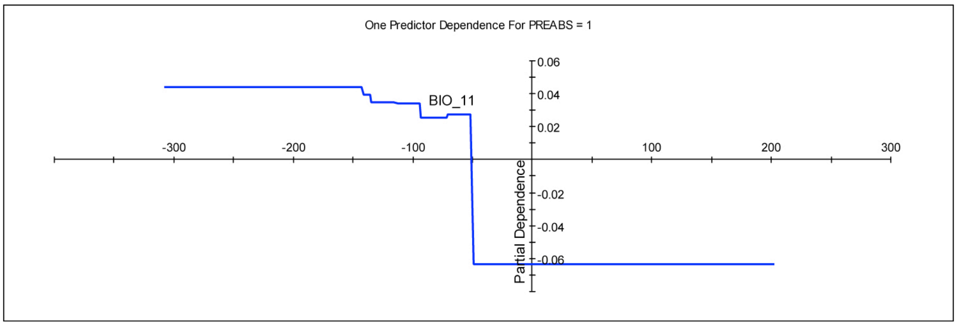

13. Bio_12

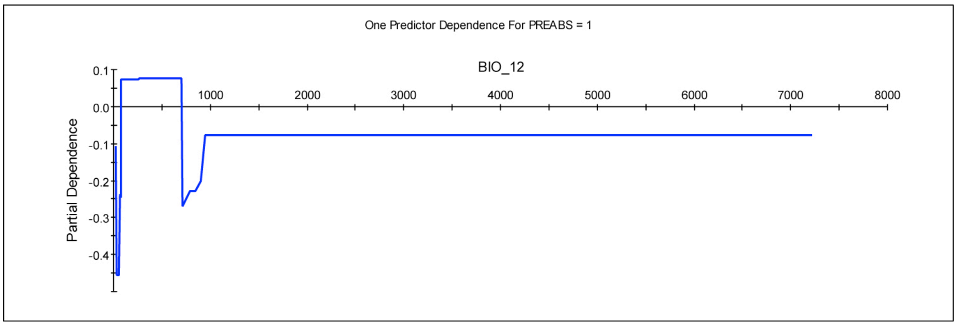

14. Bio_13

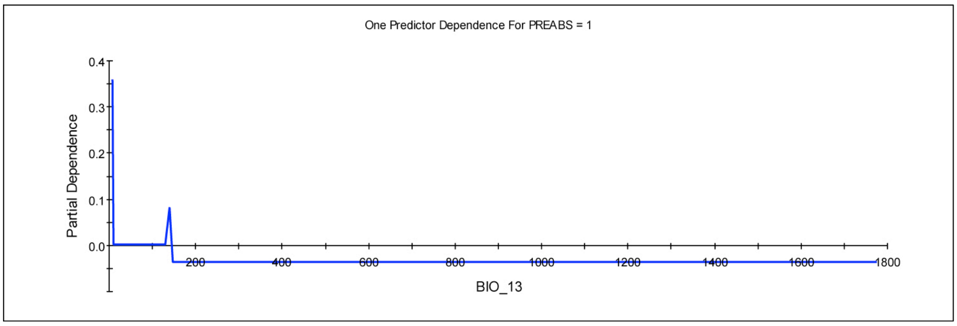

15. Bio_15

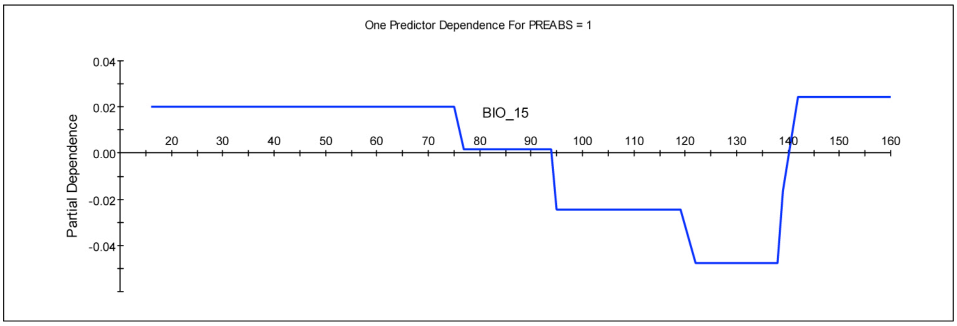

16. Bio_16

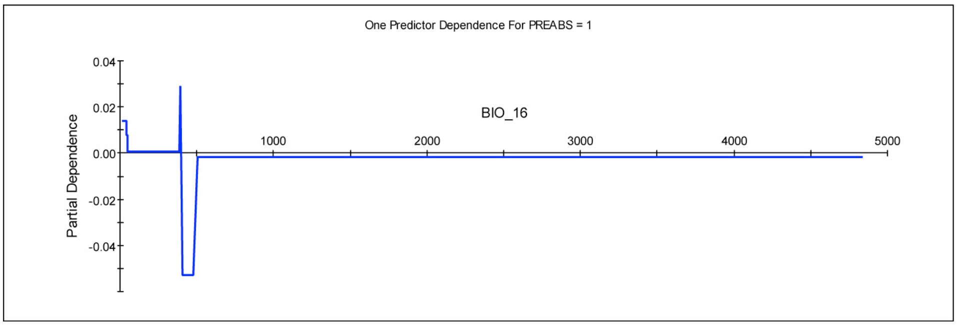

17. Bio_17

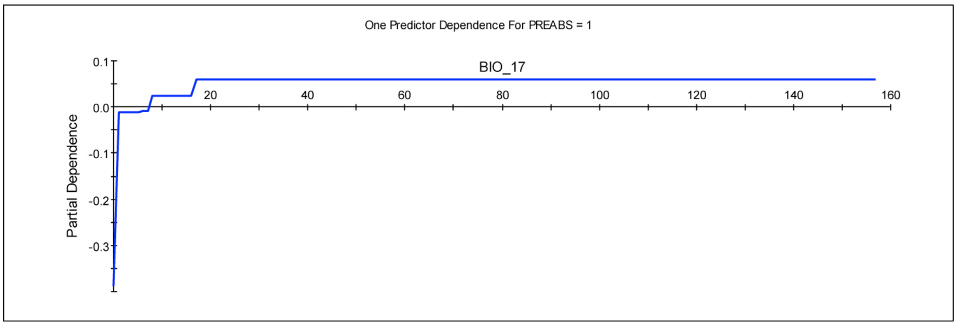

18. Bio_18

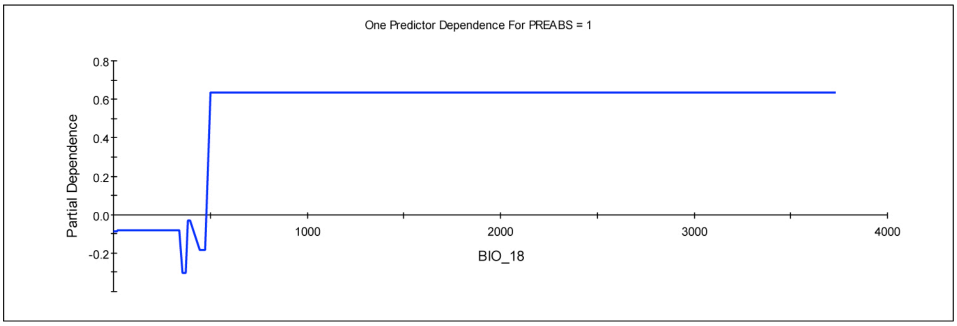

19. Bio_19

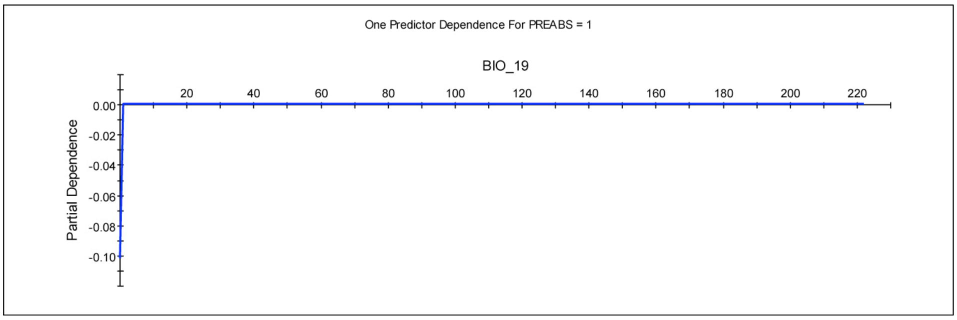

20. Discsln

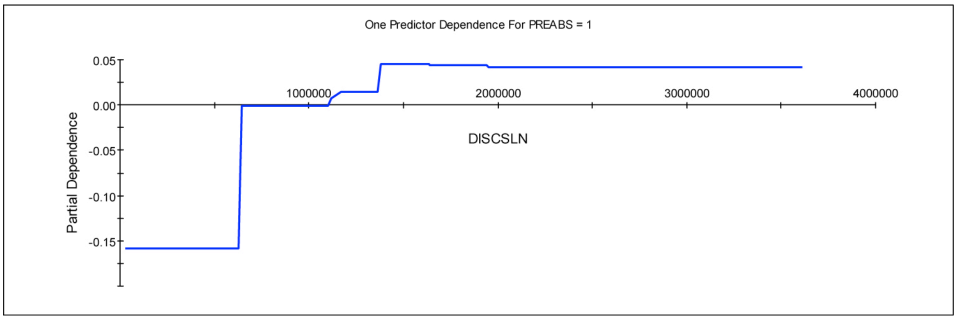

21. Dislake

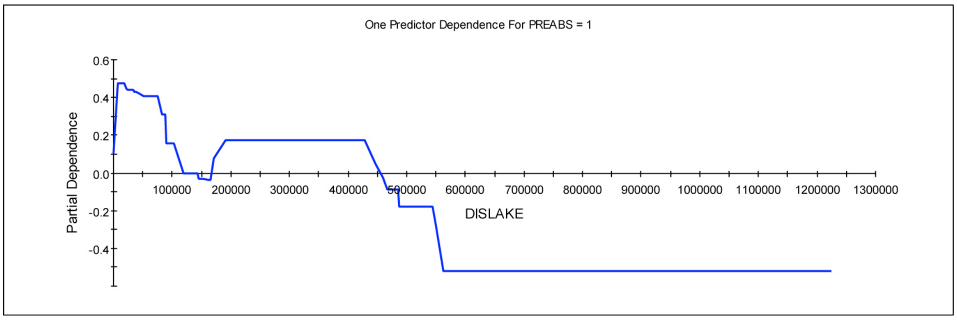

22. Disrard

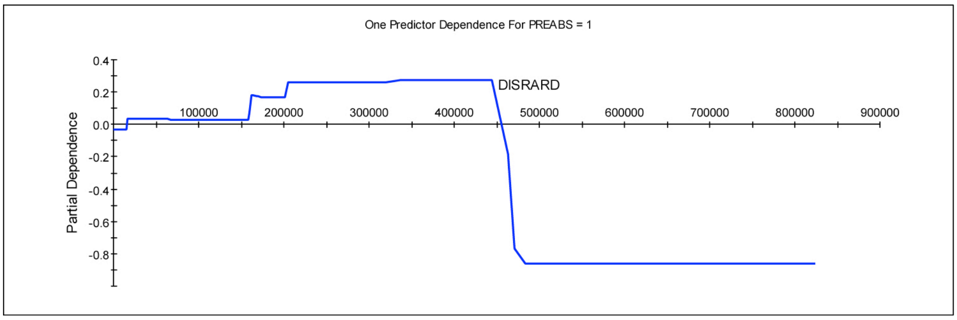

23. Disriver

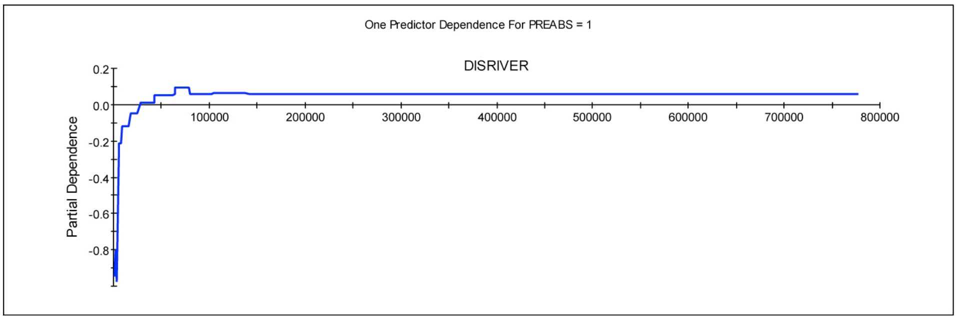

24. Disroad

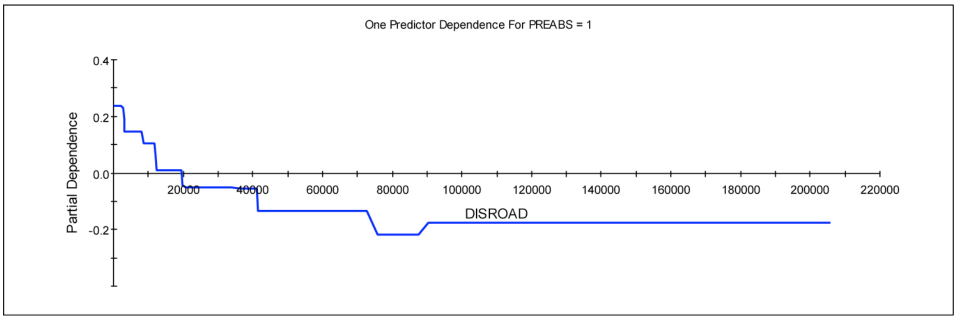

25. Dissettle

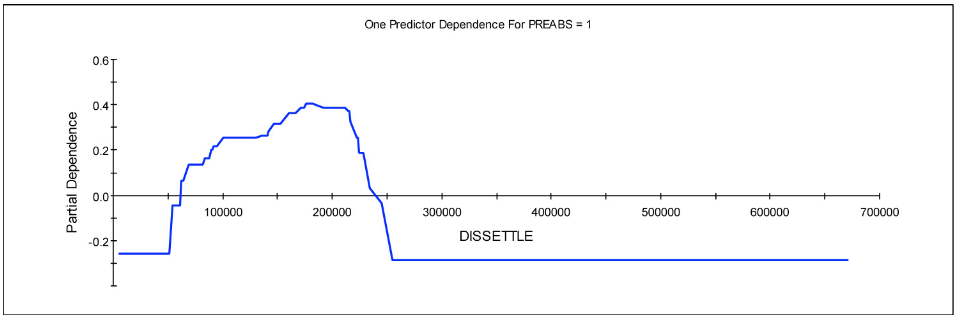

26. Landcv (land cover class)

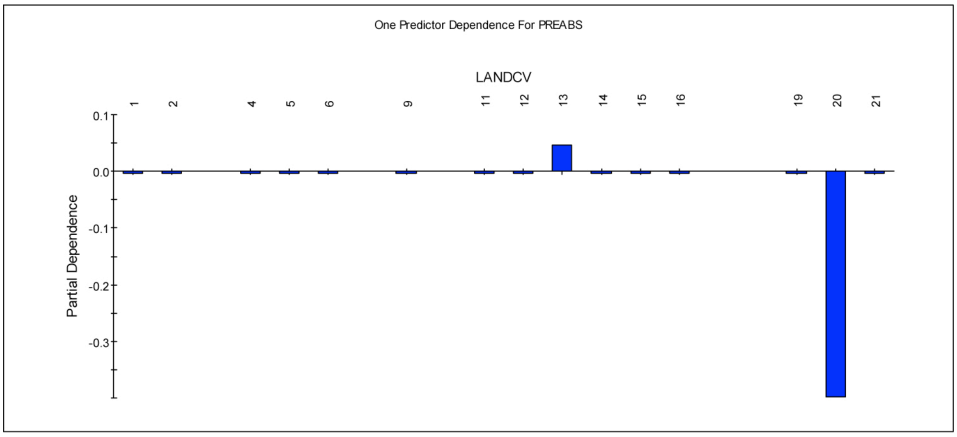

(Detailed land cover information was presented in Table S2.)

Supplementary Table S1. Environmental variables and importance rankings.

All the variable layers are downloaded from online Open Access: WorldClim, http://www.worldclim.org/; European Space Agency, http://www.esa-landcover-cci.org/; Natural Earth, http://www.naturalearthdata.com/.

Rank	Variable	Description	Score	Project Source	Resolution
1	Altitude	Height above Sea Level (m)	100.000	Derived from Altitude, see WorldClim	1000 m
2	Bio_5	Max Temperature of Warmest Month (°C)	99.273	WorldClim	1000 m
3	Bio_4	Temperature Seasonality (standard deviation *100°C)	50.000	WorldClim	1000 m
4	Landcv	Land Cover Class	45.092	European Space Agency	300 m
5	Bio_9	Mean Temperature of Driest Quarter (°C)	37.520	WorldClim	1000 m
6	Bio_1	Annual Mean Temperature (°C)	29.557	WorldClim	1000 m
7	Disrard	Distance to Railways (m)	24.486	Rail road layer from Natural Earth	1000 m
8	Bio_7	Temperature Annual Range (BIO5-BIO6) (°C)	22.978	WorldClim	1000 m
9	Bio_8	Mean Temperature of Wettest Quarter (°C)	19.165	WorldClim	1000 m
10	Bio_6	Min Temperature of Coldest Month (°C)	17.141	WorldClim	1000 m
11	Discsln	Distance to Coastline (m)	14.188	Coastline layer from Natural Earth	1000 m
12	Dissettle	Distance to Settlements (m)	11.593	Settle layer from Natural Earth	1000 m
13	Disroad	Distance to Roads (m)	10.724	Road layer from Natural Earth	1000 m
14	Dislake	Distance to Lakes (m)	9.476	Lake layer from Natural Earth	1000 m
15	Bio_11	Mean Temperature of Coldest Quarter (°C)	9.035	WorldClim	1000 m
16	Bio_16	Precipitation of Wettest Quarter (mm)	8.705	WorldClim	1000 m
17	Bio_2	Mean Diurnal Range (max temp - min temp)(°C)	6.595	WorldClim	1000 m
18	Bio_19	Precipitation of Coldest Quarter (mm)	5.941	WorldClim	1000 m
19	Bio_12	Annual Precipitation (mm)	5.667	WorldClim	1000 m
20	Disriver	Distance to Rivers (m)	5.197	River layer from Natural Earth	1000 m

21	Bio_18	Precipitation of Warmest Quarter (mm)	5.167	WorldClim	1000 m
22	Bio_3	Isothermality (BIO2/BIO7) (*100°C)	4.468	WorldClim	1000 m
23	Bio_13	Precipitation of Wettest Month (mm)	4.101	WorldClim	1000 m
24	Slope	Tangent of the Angle of Surfaces to the Horizontal	2.544	Derived from Altitude	1000 m
25	Aspect	Compass Direction of Slopes (°)	0.834	Derived from Altitude	1000 m
26	Bio_15	Precipitation Seasonality (mm)	0.594	WorldClim	1000 m
27	Bio_17	Precipitation of Driest Quarter (mm)	0.301	WorldClim	1000 m
28	Bio_14	Precipitation of Driest Month (mm)	0.050	WorldClim	1000 m
29	Bio_10	Mean Temperature of Warmest Quarter (°C)	0.000	WorldClim	1000 m

Supplementary Table S2. Detail for the predictor "Landcv" (Land cover class).
Downloaded from European Space Agency, see http://www.esa-landcover-cci.org/ for more detail.

**Num.**	**Land Cover Class Description**
1	Tree Cover, broadleaved, evergreen<br>*LCCS >15% tree cover, tree height >3m*<br>(Examples of sub-classes at regional level* :<br>*closed > 40% tree cove; open 15-40% tree cover)*
2	Tree Cover, broadleaved, deciduous, closed
3	Tree Cover, broadleaved, deciduous, open<br>*(open 15-40% tree cover)*
4	Tree Cover, needle-leaved, evergreen
5	Tree Cover, needle-leaved, deciduous
6	Tree Cover, mixed leaf type
7	Tree Cover, regularly flooded, fresh water (& brackish)
8	Tree Cover, regularly flooded, saline water,<br>(daily variation of water level)
9	Mosaic: Tree Cover / Other natural vegetation
10	Tree Cover, burnt
11	Shrub Cover, closed-open, evergreen<br>(Examples of sub-classes at reg. level *: (i) sparse tree layer)
12	Shrub Cover, closed-open, deciduous<br>(Examples of sub-classes at reg. level *: (i) sparse tree layer)
13	Herbaceous Cover, closed-open<br>(Examples of sub-classes at regional level *:<br>(i) natural, (ii) pasture, (iii) sparse trees or shrubs)
14	Sparse herbaceous or sparse shrub cover
15	Regularly flooded shrub and/or herbaceous cover
16	Cultivated and managed areas<br>(Examples of sub-classes at reg. level *:<br>(i) terrestrial; (ii) aquatic (=flooded during cultivation), and under terrestrial: (iii) tree crop & shrubs (perennial), (iv) herbaceous crops (annual), non-irrigated, (v) herbaceous crops (annual), irrigated)
17	Mosaic: Cropland / Tree Cover / Other natural vegetation
18	Mosaic: Cropland / Shrub and/or grass cover
19	Bare Areas
20	Water Bodies (natural & artificial)
21	Snow and Ice (natural & artificial)
22	Artificial surfaces and associated areas

Supplementary Table S3. Threshold table for Random Forest model

Generated by SPM software automatically; a represents true positive (or presences), b represents false positive (or presences), c represents false negatives (or absences), d represents true negative (or absences), n (=a+b+c+d) is the total number of presence and absence points.

	a	b	c	d	n
0.00	183	18300	0	0	18483
0.01	182	3943	1	14357	18483
0.02	182	2678	1	15622	18483
0.03	182	2515	1	15785	18483
0.04	182	2477	1	15823	18483
0.05	182	2456	1	15844	18483
0.06	182	2439	1	15861	18483
0.07	182	2426	1	15874	18483
0.08	182	2407	1	15893	18483
0.09	182	2371	1	15929	18483
0.10	182	2341	1	15959	18483
0.11	182	2328	1	15972	18483
0.12	182	2305	1	15995	18483
0.13	182	2244	1	16056	18483
0.14	182	2207	1	16093	18483
0.15	182	2197	1	16103	18483
0.16	182	2179	1	16121	18483
0.17	182	2157	1	16143	18483
0.18	182	2150	1	16150	18483
0.19	182	2146	1	16154	18483
0.20	182	2139	1	16161	18483
0.21	182	2006	1	16294	18483
0.22	182	1697	1	16603	18483
0.23	182	1486	1	16814	18483
0.24	182	1434	1	16866	18483
0.25	182	1414	1	16886	18483
0.26	182	1408	1	16892	18483
0.27	182	1381	1	16919	18483
0.28	182	1363	1	16937	18483
0.29	182	1345	1	16955	18483
0.30	182	1315	1	16985	18483
0.31	182	1292	1	17008	18483
0.32	182	1278	1	17022	18483
0.33	182	1261	1	17039	18483
0.34	182	1243	1	17057	18483
0.35	182	1198	1	17102	18483
0.36	181	1138	2	17162	18483

0.37	181	1090	2	17210	18483
0.38	181	1064	2	17236	18483
0.39	181	1047	2	17253	18483
0.40	180	1024	3	17276	18483
0.41	180	1002	3	17298	18483
0.42	180	992	3	17308	18483
0.43	180	971	3	17329	18483
0.44	180	954	3	17346	18483
0.45	180	924	3	17376	18483
0.46	180	891	3	17409	18483
0.47	180	876	3	17424	18483
0.48	180	856	3	17444	18483
0.49	180	836	3	17464	18483
0.50	180	821	3	17479	18483
0.51	180	801	3	17499	18483
0.52	180	781	3	17519	18483
0.53	180	761	3	17539	18483
0.54	180	739	3	17561	18483
0.55	180	724	3	17576	18483
0.56	180	711	3	17589	18483
0.57	180	691	3	17609	18483
0.58	180	671	3	17629	18483
0.59	179	657	4	17643	18483
0.60	179	642	4	17658	18483
0.61	178	632	5	17668	18483
0.62	178	615	5	17685	18483
0.63	178	597	5	17703	18483
0.64	178	578	5	17722	18483
0.65	178	564	5	17736	18483
0.66	178	548	5	17752	18483
0.67	178	530	5	17770	18483
0.68	177	511	6	17789	18483
0.69	176	497	7	17803	18483
0.70	176	481	7	17819	18483
0.71	176	463	7	17837	18483
0.72	176	451	7	17849	18483
0.73	176	431	7	17869	18483
0.74	176	414	7	17886	18483
0.75	176	398	7	17902	18483
0.76	176	385	7	17915	18483
0.77	176	369	7	17931	18483
0.78	175	353	8	17947	18483
0.79	175	337	8	17963	18483

0.80	174	325	9	17975	18483
0.81	174	308	9	17992	18483
0.82	173	291	10	18009	18483
0.83	173	277	10	18023	18483
0.84	173	261	10	18039	18483
0.85	173	247	10	18053	18483
0.86	173	228	10	18072	18483
0.87	172	215	11	18085	18483
0.88	170	194	13	18106	18483
0.89	168	187	15	18113	18483
0.90	168	171	15	18129	18483
0.91	167	153	16	18147	18483
0.92	166	137	17	18163	18483
0.93	166	113	17	18187	18483
0.94	165	100	18	18200	18483
0.95	160	76	23	18224	18483
0.96	155	61	28	18239	18483
0.97	153	35	30	18265	18483
0.98	142	16	41	18284	18483
0.99	128	7	55	18293	18483
1.00	0	0	183	18300	18483

Supplementary Table S4. Detailed statistics for conservation overlay analysis.

High or low HII area was inferred by the threshold of HII=10; protected predicted breeding area is the predicted presence area (RIO≥0.58) within the protected areas; conservation gaps are the high HII areas but located outside the protected areas. Area statistics were calculated in ArcGIS 10.1 (ERSI co., USA).

**GIS Layer**	**Area (km^2)**	**Percentage (%)**
HII 0-4 (low)	231,118.78	27.46
HII 5-9 (low)	299,222.20	35.55
HII 10-14 (high)	233,804.56	27.78
HII 15-19 (high)	66,859.27	7.94
HII 20-24 (high)	8,826.83	1.05
HII 25-29 (high)	1,247.01	0.15
HII 30-34 (high)	570.91	0.07
HII 35-39 (high)	36.76	0.00
HII 40-44 (high)	36.36	0.00
HII 45-49 (high)	16.45	0.00
HII 50-54 (high)	5.73	0.00
HII 55-59 (high)	3.60	0.00
HII 60-64 (high)	1.23	0.00
Protected Predicted Breeding Area	198,976.47	23.64
Conservation Gap	225,081.82	26.74
Predicted Breeding Area	841,749.70	100.00
